# Supplementary material for: Implementing organicity investigations in early psychosis: Spreading expertise
Source: PLoS One. 2021 Jun 10;16(6):e0252610. doi: 10.1371/journal.pone.0252610 (PMC8191881; doi:10.1371/journal.pone.0252610)
Supplement: S1 Table — (DOCX) [file pone.0252610.s001.docx]

# S1 Table: Analysis details

| Themes | Categories | Quotes |
| --- | --- | --- |
| **The daily effects of OI** | | |
| Advantages of OI | A relational tool for families | When you explain that it's to be certain that there is nothing else that might explain the symptoms.… it’s reassuring for [the parents]. (1)  Although it's not necessarily less serious, but for the public, it's easier to say "He has Wilson's disease than he is developing schizophrenia". (1) |
|  | A technical tool for doctors | To start on a serene foundation… If I see a youth who has had a first episode, the work-up was a little light or they didn't look in detail, I will say to myself: was that really a psychiatric episode? That is after all the foundation of working with children and adolescents. (2)  Psychiatry, it's fuzzy, it’s vague, it’s invisible …. There's a reassuring material aspect to a work-up, you see it on a computer, the blood test you see it, the brain MRI you see it.… It's based on a physical thing. (2) |
| Limitations of OI | Short-term avoidance of psychiatry | So now major worries .… "Isn't this the start of a nightmare because my son is going to become schizophrenic? Is he going to be able to have a normal life?" … At the same time, they are happy that there's nothing, but, ok, it's psychiatric. (2) |
|  | Unwelcome tests for the patients | We tried, one after the other, but we couldn't do anything. She didn't want this spinal tap. I was willing to have it done under general anesthesia if necessary…. She started to say: you want to put me to sleep so you can take away my baby. (4) |
|  | For psychiatrists, an alienating and constraining process | Maybe we should have thought of it, by looking, examining, a little better? But even as a pediatrician, even as a psychiatrist, we certainly don't have any etiological finesse in dermatology. Maybe a dermatologist would have said, "look, this should be checked out". (7)  Remember exactly what mechanism and why we're looking for it, it's complicated. So, intellectually, I say to myself, "good, I'll do it" but it's not … super fulfilling. (2) |
|  | A constraining procedure for physicians | The practical side, it's already just that I want to test for an MTHFR mutation, the order form I get it where? What do I do? At the [EC] it's a big machine, there's a department head, there are three residents in the department. I'm all alone. (2) |
| **To transform: Implementing OI as a young psychiatrist** | | |
| Young experts joining an aging institution | The duty of medical progress | Child and adolescent psychiatry is a medical specialty, and I think it's one of our jobs to know how to suspect organic causes, to know how to order organic and neurometabolic work-ups. (10) |
|  | A driving force for implementation | "My department head was a little surprised at the whole procedure and told me that it's absolutely necessary for me to train them. (8)  I’ve converted quite a few, in giving out different metabolic work-ups. I’ve spread the gospel [laughs]. (1) |
|  | Disseminating knowledge tactfully | You have to know how to bring them around! In our specialty, you can often run into a somewhat hermetic.… I had said, "maybe, nonetheless; I find it weird that … there are neurologic diseases that present a little like that". (10)  There are more political interfaces that don't simplify the fact of being able to apply the practices of one department to another. I was completely free to do what I wanted, but on the other hand, I was alone. (5) |
| Reshaping the use of OI | Perseverance | [The examinations] they… recommended during … training at [the expert center]. (1) |
|  | Renunciation | It's sort of a risky business… It's good to ask for advice, that seems the most reasonable to me. (7)  I did it a little at the start of my residency, saying to myself: good, we'll see if we keep finding things? I didn't continue because I didn't find any folate deficiencies. (5) |
|  | Pragmatic patchwork | I do a genetic work-up very rarely because I think it's really a geneticist's job and that I really don't know how to give the results, I don't know how to say what it means. (8)  You have to differentiate what is the easiest and the least expensive. And what's most complicated, most technical, it needs to be done at a university hospital. (7) |
| Seeking external support | Referral | When I think it might be organic, I refer the patients [to the EC] for a work-up. … You have to know how to stay humble. I prefer to ask the advice of someone who knows what they're doing. But you don't want to be taken for an idiot…by colleagues at the [EC] who think you know really nothing about nothing […] It's unpleasant to send a patient for an opinion and instead of discussing it, the colleague tells the patient that you were wrong. (7) |
|  | Partnership | When there are things that are very interconnected, genetic-neuro-psychiatric, I think it's good to be able to make decisions jointly and discuss it.… Then you don't have to have all the weight on your own shoulders. (7)  The conversation with the professor, it serves two purposes: get his opinion about the prescriptions and so the mother can accept the decisions you're making. Because, "Professor at the expert center", so. (11)  In the [ECs] they love that. I remember one patient, they called us saying: "keep her for us". When it's a combination with organic, because they had a study underway. When you can write articles, that's interesting. (13) |
|  | Seeking accurate somatic assessments | Our colleagues who deal with somatic diseases, I think they don't care at all [whispered]. When we have a question about an organic aspect, we're the ones who are interested. (4)  When neurologists and especially radiologists see: 'MRI for psychiatric disease' and 'rule out organic cause', don't they tend to say very quickly that the MRI is normal? (7) |
|  | Experiencing loneliness | When you have to write back to the pediatrician, I don't know, question her? Or who do you ask the questions? Finally, who do you call back? The neurologist she saw [at the EC]? Finally… (11) |
| **To be transformed: changes in practices, as psychiatrists gain experience** | | |
| The resident on a mission | Professional ethics: a central driving force | There is a work of connection, a work of organization, a loss of time. As a doctor, I have a best-efforts obligation and for me, it's part of my obligations to my patient. (3) |
| The rise of an inner conflict | Service protocols and compromises to adjust to organizational constraints | I'm not certain we could do all that correctly. (7)  The question that was asked when I arrived was, are we going to formalize it? It's been 10 years and we still haven't. So we cobble things together. It's not consistent. (3)  In choosing examinations, how do you prioritize them? Already, the most practical: what you can order at the hospital, what you can do onsite. (2) |
| Senior practitioners who give up OI | Disavowal of their own ideals and betrayal of their teachers | It hurts a little, because it doesn't correspond to what we said we'd do, what we'd be. If I discussed it with my old thesis director, he would say 'How did you manage to get there? You're just doing it halfway." That's it… [laughs]. It's not my style to ignore that. (11) |
